# Molecular Aspects of the Functioning of Pathogenic Bacteria Biofilm Based on *Quorum Sensing* (QS) Signal-Response System and Innovative Non-Antibiotic Strategies for Their Elimination

**DOI:** 10.3390/ijms25052655

**Published:** 2024-02-24

**Authors:** Edyta Juszczuk-Kubiak

**Affiliations:** Laboratory of Biotechnology and Molecular Engineering, Department of Microbiology, Prof. Wacław Dąbrowski Institute of Agricultural and Food Biotechnology—State Research Institute, Rakowiecka 36 Street, 02-532 Warsaw, Poland; edyta.juszczuk-kubiak@ibprs.pl

**Keywords:** quorum sensing (QS), autoinducers (AIs), bacterial pathogens, antimicrobial resistance (AMR), biofilm formation, quorum quenching (QQ), innovative antibiofilm strategies

## Abstract

One of the key mechanisms enabling bacterial cells to create biofilms and regulate crucial life functions in a global and highly synchronized way is a bacterial communication system called quorum sensing (QS). QS is a bacterial cell-to-cell communication process that depends on the bacterial population density and is mediated by small signalling molecules called autoinducers (AIs). In bacteria, QS controls the biofilm formation through the global regulation of gene expression involved in the extracellular polymeric matrix (EPS) synthesis, virulence factor production, stress tolerance and metabolic adaptation. Forming biofilm is one of the crucial mechanisms of bacterial antimicrobial resistance (AMR). A common feature of human pathogens is the ability to form biofilm, which poses a serious medical issue due to their high susceptibility to traditional antibiotics. Because QS is associated with virulence and biofilm formation, there is a belief that inhibition of QS activity called quorum quenching (QQ) may provide alternative therapeutic methods for treating microbial infections. This review summarises recent progress in biofilm research, focusing on the mechanisms by which biofilms, especially those formed by pathogenic bacteria, become resistant to antibiotic treatment. Subsequently, a potential alternative approach to QS inhibition highlighting innovative non-antibiotic strategies to control AMR and biofilm formation of pathogenic bacteria has been discussed.

## 1. Introduction

Bacterial processes, such as biofilm formation, secretion of the virulence factor, bioluminescence, production of antibiotics, secondary metabolites, sporulation, apoptosis, and horizontal gene transfer (HGT) ability, are necessary for the functioning of these microorganisms in the external environment [1,2]. However, these metabolic processes are ineffective if they occur during the planktonic growth phase of individual bacterial cells [3,4]. We know, however, that bacteria have successfully developed an “intelligent” system of cell cooperation, communication, and control mechanisms to survive in the unfavourable conditions of the surrounding environment [5,6].

How are bacteria doing? Through quorum sensing (QS), bacteria synchronously control the global gene expression in response to changes in cell density and species complexity [7,8]. Detecting the quorum allows bacteria to switch between two different gene expression programs. The first (1), preferred at low cell density (LCD), promotes individual antisocial behaviour. The second (2), favoured at high cell density (HCD), promotes community behaviour, also known as group behaviour [9,10,11,12]. Adapting to environmental changes requires the bacterial community to integrate external signals and coordinate intracellular responses based on global regulatory networks. The basic processes related to detecting and reacting to changes in the number of bacterial cells are analogous in all known bacterial quorum detection systems [10,11,13,14]. First, signal molecules called autoinducers (AIs) are synthesized intracellularly. Second, these molecules are either passively released or secreted outside the cellular environment. As the number of cells in the population increases, so does the extracellular autoinducer concentration. Third, when signalling molecules accumulate above the minimum threshold required for detection, their cognate receptors bind to the autoinducer and trigger a signalling cascade that changes gene expression within the bacterial population [11,15,16]. Thus, quorum detection enables the coordinated functioning of the bacterial cell population, thereby increasing the chance of survival in adverse environmental conditions [11].

It is well known that bacteria form a biofilm under the control of the QS system [13,15,17,18,19]. Several excellent reviews discuss how microorganisms develop pathogenic biofilms and their protective mechanisms against antibiotics, antimicrobial agents, and host innate immunity [4,20,21,22]. In 2017, the World Health Organization (WHO) prepared a list of bacterial strains (ESKAPE) like *Enterococcus faecium*, *Staphylococcus aureus*, *Klebsiella pneumoniae*, *Acinetobacter baumanii*, *Pseudomonas aeruginosa*, and *Enterobacter* spp., which are developed through several molecular mechanisms of antimicrobial resistance (AMR), making them ineffective in traditional antibiotic therapy [23,24]. These pathogens are responsible for nearly 80% of hospital-acquired infections, particularly in critically ill patients, due to their capacity for biofilm formation [25,26]. For instance, previous studies have shown that the pathogenicity of *P. aeruginosa* is closely related to the biofilm [27]. *E. faecium* and *S. aureus* resisted various antibiotics, such as vancomycin and fluoroquinolones [28].

Currently, antibiotics are still a significant treatment for pathogens infections. However, biofilms, being a barrier around bacterial cells, reduce the receptivity of bacteria to conventional antibiotics, leading to persistent infections. For instance, Hoiby et al. [29] observed that biofilm bacteria increase antibiotic resistance by about 1000 fold. The intensive development of bacterial resistance to antimicrobial agents is currently a new, major threat to public health care [24,30]. Therefore, discovering alternative non-antibiotic strategies for inhibiting bacterial biofilms is urgent due to biofilm resistance to already-used antimicrobial agents [31]. Because QS controls a broad spectrum of phenotypes, including virulence and biofilm formation, inhibition of QS may provide alternative therapeutic methods for treating microbial infections [32]. The strategy of blocking the QS system and inhibiting virulence factor production is called quorum quenching (QQ) [13,33,34]. QQ is a promising non-antibiotic alternative for the treatment of a broad range of pathogenic bacterial infections, including QQ enzymes, which inactivate QS signals, and QS inhibitors (QSIs), which chemically disrupt QS pathways via inhibition of signal receptors [33,35]. Moreover, several other innovative therapeutic strategies, like antimicrobial peptides [36], antibodies [37], nanoparticles [38], probiotics [39], and phage therapy [40], as well as precision genome targeting [41], aimed at effectively eradicating biofilm-related infections, are currently under investigation. Despite tremendous progress in antibiotic-resistant mechanisms and corresponding strategies to override resistance, biofilm-associated infections remain a considerable challenge.

Given the important role of quorum sensing (QS) in biofilm formation, this review summarised recent progress in biofilm research, focusing on the mechanisms by which biofilms, especially those formed by pathogenic bacteria, become resistant to antibiotic treatment. In the first part of the review, the role of main QS systems in the global expression regulation of multiple genes involved in the pathogenicity of the biofilm-forming bacteria has been systematized. The second part of the review focused on recent developments in antibiofilm strategies by disrupting the quorum sensing system, which is critical for biofilm formation, and summarised different classes of antimicrobial compounds to control biofilm formation.

## 2. Genetic Modules and Their Homologues as Regulatory Networks Detecting QS

The QS system presented in Gram-positive and Gram-negative bacteria is involved in biofilm formation, bacterial adhesion, host colonization, and expression of many virulence factors [17]. Moreover, several studies have QS’s crucial role in gut microbiota–host cell interaction [31]. QS regulates gene expression dependent on cell population density, facilitated by small signalling molecules known as autoinducers (AIs) [11,42]. Therefore, AIs are called “hormone-like molecules”, whereas the biofilm is considered a multicellular organism [11,16,43]. The AIs are products of the specific genes, and then after modification, they diffuse freely across the cell membranes or are actively transported out of the cell [13,14,44]. Once the concentration of secreted Al molecules has reached a threshold level, they are detected by cognate sensor proteins. These proteins either transduce the signal to downstream transcriptional regulators or function as transcriptional regulators to mediate changes in global gene expression [15,18,45].

### 2.1. QS in Gram-Negative Bacteria

#### 2.1.1. AHL Signalling

The primary signalling molecules in Gram-negative bacteria are homoserine lactones (AHLs), called acyl-homoserine lactones, known as AI-1 autoinducers [16]. AI-1 is used in intraspecific communication of biofilm-forming bacteria [10], although some bacteria can detect competing bacterial species in the surrounding environment [14]. In Gram-negative bacteria, the QS based on AHLs plays a vital role in regulating global gene expression in response to the density of bacterial cells [16]. This type of QS signal is found in more than 70 species of bacteria, most of which are pathogens [12,46,47].

**Figure 1:**
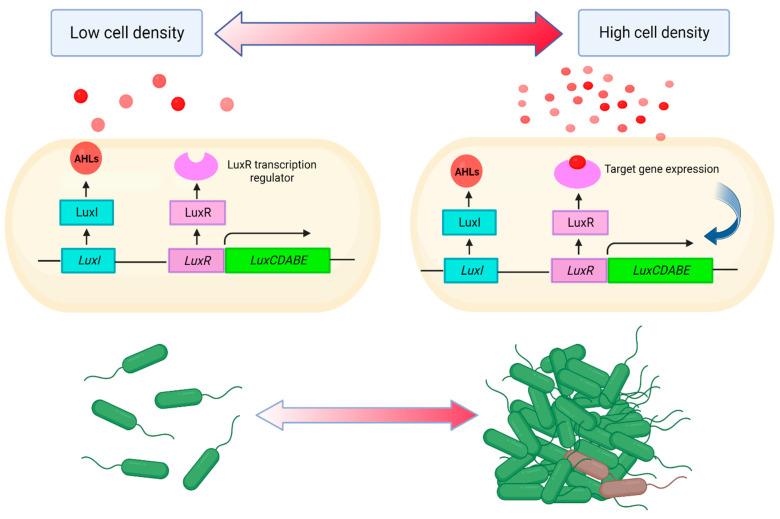
General mechanism of QS in Gram-negative bacteria scheme of activation of the *lux* operon by *luxI* and *luxR* in *Vibrio fischeri*. The autoinducers (3OC6-HSL: red dots), produced by LuxI, diffuse through the cell membrane into the growth medium at low cell density. As the cell growth continues, the autoinducers in the medium accumulate in a confined environment. A very low intensity of light can be detected. When enough autoinducers have accumulated in the medium, they can re-enter the cell, directly binding the LuxR protein to activate *luxICDABEG* expression. High levels of autoinducers activate the luminescent system of *A. fischeri.* High-intensity light can be detected. The figure was created with BioRender (https://biorender.com/, 4 February 2023).

The best-known AHL-mediated QS mechanism in Gram-negative bacteria is the LuxI/LuxR system, which was described for the first time in *V. fischeri* (Figure 1). LuxI-type proteins are responsible for the synthesis of AI-1, predominantly 3-oxo-hexanoyl-l-homoserine lactone (3OC_6_-HSL), which passively penetrates the cell membrane and transmits a signal transmission between cells [6,7]. The N-terminal domain of LuxR protein recognises and binds AI-1. In contrast, the C-terminal domain, via conserved helix-turn-helix motif, interacts with the promoter of multiple target genes in the region of their palindromic sequence (lux-box), located about 40 bp upstream of the ATG codon [11,16,42]. After reaching the threshold, AHLs and LuxR form the LuxR–AHLs complex, which recognises the “*lux* box” of *luxI* to promote the *luxI* transcription, creating a positive feedback loop [48,49,50].

Similar LuxI/LuxR-type homologues have been identified in other Gram-negative bacterial species. In *Pseudomonas aeruginosa*, two AHL-depended systems, namely LasR/LasI and RhlR/RhlI, responsible for the synthesis of the N-(3-oxo-dodecanoyl)-L-homoserine lactone (3OC_12_-HSL) and N-butanoyl-L-homoserine lactone (C_4_-AHL) were described [51,52,53]. Both systems are key expression regulators of many virulence factors, including elastase (lasB), proteases (lasA, aprA), exotoxin A (toxA), rhamnolipids (rhlAB), pyocyanin (phzABCDEFG, phzM), and lectins (lecA) [54]. In turn, in *Serratia*, several different LuxI/LuxR-type systems, such as SwrI/SwrR (*Serratia liquefaciens* MG1) [55,56], SmaI/SmaR (*Serratia* sp. Strain ATCC 39006) [57], SprI/SprR (*Serratia proteamaculans*) [58], and SpnI/SpnR (*Serratia marcescens* SS-1) have been identified [59]. In *S. marcescens* SS-1, SpnI protein synthesizes at least four types of AHLs, including *N*-3-oxohexanoyl-homoserine lactone (3OC_8_-HSL), *N*-hexanoyl-homoserine lactone (C_6_-HSL), *N*-heptanoyl-homoserine lactone (C_7_-HSL), and *N*-octanoyl-homoserine lactone (C8-HSL) [60]. In contrast to most other LuxR homologues, SpnR acts as a negative regulator and is derepressed by 3OC_6_-HSL [55,58]. The SpnI/SpnR is involved in the prodigiosin, rhamnolipid, and nuclease synthesis [55,59]. In addition, *spnI/spnR* genes might be located in a mobile DNA region and have been involved in HGT. Wei et al. [55] showed that the SpnR/SpnI carried by a Tn3 transposon in *S. marcescens* SS-1 can be moved between plasmids and chromosomes of this species and *E. coli* coexisting in the same environment. The acquisition of the SpnR/SpnI system by *E. coli*, which does not synthesize AI-1 in the natural environment, significantly changes the metabolism of this species.

*Escherichia coli*, *Salmonella*, *Klebsiella*, *Shigella*, and *Enterobacter* encode SdiA, a transcription factor of the LuxR family that regulates gene expression in response to AHLs produced by other bacterial species [61]. SdiA was found to detect a wide range of AHLs, including 3OC_8_-HSL and *N*-3-oxo-decanoyl-L-homoserine lactone (3OC_10_-HSL), *N*-hexanoyl-L-homoserine lactone (C_6_-HSL), and *N*-octanoyl-L-homoserine lactone (C_8_-HSL) [62,63]. In *Salmonella*, the *sdiA* regulates the *rck* expression, which is involved in the adhesion and invasion of host epithelial cells [64]. The *rck* is located on the virulence plasmid of pRST98 [65]. In *Salmonella* strains carrying pRST98, AHLs increase *rck* expression, enhancing bacterial adherence, serum resistance, and biofilm formation [65]. In enterohemorrhagic *Escherichia coli* (EHEC), *Enterobacter*, and *E. coli* K-12 BW25113, *sdiA* participates in the regulation of several virulence factors such as curli production, adhesion on epithelial cells, and biofilm formation [61,66,67]. The role of *sdiA* in the biofilm of pathogenic strains has been reported in several studies [61,63,68]. Lee et al. [67] showed that the isogenic *sdiA*-mutant of *E.coli* K-12 BW25113 increased biofilm formation 18 fold compared to the wild type. Similar results have been reported by Suzuki et al. [69] and Lee et al. [70]. Well-studied AHL-mediated QS systems also include the Tra/TraR in the *A. tumefaciens* [71], the EsaI/EsaR in *Pantoea stewartii* [72], as well as ExpI/ExpR in *Erwinia* [73] and TraR in *Agrobacterium* species [74].

#### 2.1.2. PQS Signalling

In *P. aeruginosa*, the third QS system is an AHL-independent system that consists of a LysR-type regulator PqsR (also known as MyfR) and the pseudomonas quinolone signal (PQS, 2-heptyl-3-hydroxy-4-quinolone) called PQS system [75]. Cell signalling of the PQS system occurs via the synthesis and modification of 4-hydroxy-2-alkyquinolines (HAQ) under the control of the transcriptional regulator PqsR. PqsR regulates the expression of the genes involved in the production of anthranilic acid and its conversion to 4-hydroxy-2-heptylquinoline (HHQ) [76]. The pqsABCDE, phnAB, and pqsH locus control the synthesis of HAQ and HHQ molecules; the *pqsA* and *pqsBCD* genes encode the ligase and synthases involved in the precursor HHQ synthesis. HHQ, following subsequent modifications via the action of the FAD-dependent mono-oxygenase encoded by pqsH, is converted to PQS [77,78]. Recent studies suggest the role of *pqsE* in thioesterase TesB synthesis, which is involved in the HHQ synthesis pathway [79]. The resulting PQS and HHQ autoinducers, after exceeding the critical threshold required for QS induction, bind and activate *pqsR* and *pqsH* mRNA transcription under the control of LasR. PQS and HHQ play dual roles as PqsR ligands and as extracellular signalling molecules for the *pqsR* regulon, although there are differences in their biological properties [77,78,80,81]. Diffusion of hydrophobic PQS into the biofilm matrix occurs via the secretion of small membrane vesicles (MVs) [77,78]. In the *P. aeruginosa* genome, the PQS-PqsR complex controls the expression of over 12% of genes involved, among others, in the biosynthesis of rhamnolipids, pyocyanin, elastase, iron acquisition, resistance to oxidative stress, and biofilm formation [80]. PQS signalling creates a network of connections with the PqsR, Las, and Rhl systems to regulate the production of several common factors involved in biofilm formation, such as LecA and siderophores [82]. The factor controlled by the PQS/PqsR system is extracellular DNA (eDNA), which is essential for forming stable and mature biofilms [83]. Accumulation of 2-n-heptyl-4-hydroxyquinoline N-oxide (HQNO), whose production is controlled by PQS signalling, leads to autolysis, eDNA release, and increased biofilm biomass [83]. In addition, Cookie et al. [84] reported that PQS induces outer membrane vesicle (OMVs) formation in *P. aeruginosa*. A significantly elevated PQS and OMV synthesis level was observed during biofilm dispersion compared to the attachment and maturation stages. Authors showed that OMVs participate in extracellular protein, lipid, and nucleotide degradation, promoting biofilm dissemination in *P. aeruginosa* infections [84,85].

#### 2.1.3. IQS Signalling

IQS has been identified as the fourth QS system in *P. aeruginosa* capable of integrating environmental stress cues with the QS network [86,87]. Several previous studies reported that synthesis of the IQS (2-(2-hydroxyphenyl) thiazole-4-carbaldehyde) is controlled by the gene cluster *ambABCDE*, while the cognate receptor is unknown [88]. For example, Lee et al. [86] reported that the disruption of LasI/LasR leads to the inhibition of the *ambBCDE* expression and reduction of the IQS synthesis. Recently, Cornelis [89] presented commentary that *ambABCDE* genes are not responsible for IQS synthesis. Results strongly suggested that IQS is aeruginaldehyde derived from the siderophore pyochelin biosynthetic pathway [88] and is produced by other *Pseudomonas*, including *P. protegens* and *Burkholderia thailandensis*, which do not have the *amb* genes cluster [88,90]. Furthermore, Rojas Murcia et al. [91] reported that the *ambBCDE* genes cluster is responsible for the biosynthesis of L-2-amino-4-methoxy-trans3-butenoic acid (AMB) but did not specify IQS in *P. aeruginosa*. Therefore, the accurate role of IQS in the QS system requires further investigation.

### 2.2. QS in Gram-Positive Bacteria

**Figure 2:**
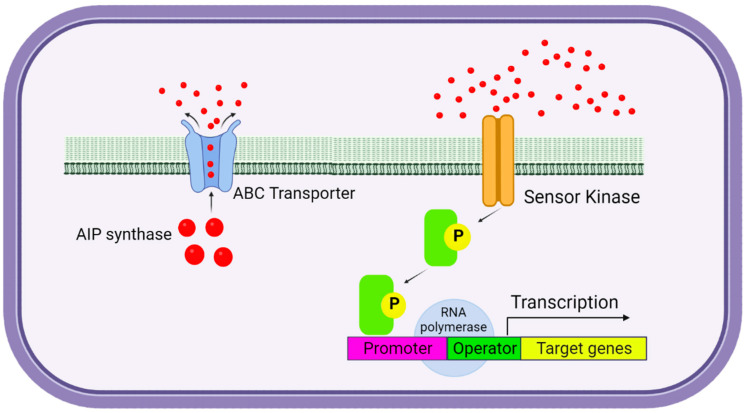
General mechanism of QS in Gram-positive bacteria. As in AHL quorum detection systems, the concentration of secreted AIP autoinducers increases with increasing cell density. Phosphorylated regulatory proteins act as DNA-binding transcription factors to modulate the expression of target genes. In many cases, the genes encoding the autoinducer precursor, the histidine kinase receptor, and the regulatory protein form an operon, and its expression is automatically induced by QS detection. This configuration produces positive feedback and accelerates the transition from LCD to HCD, a quorum-dependent mode of gene expression. The figure was created with BioRender (https://biorender.com/, 4 February 2023).

Gram-positive bacteria have developed different mechanisms of autoinducer synthesis and signal transmission from the sensor proteins of a cell to the effectors [92,93,94]. Mechanisms and proteins involved in QS in Gram-positive bacteria are best known in *Streptococcus pneumoniae*, *Bacillus subtilis*, and *Staphylococcus aureus* [95]. QS system in Gram-positive bacteria is mediated by autoinducing peptides (AIPs), which are products of the digestion of larger protein precursors [96,97]. One of the major differences between LuxI/LuxR and AIPs is the location of their cognate receptors. In the Gram-positive bacteria, LuxR-type receptors are cytoplasmic, whereas, in the Gram-positive bacteria, API receptors are membrane-bound and, as binary signalling proteins, transmit information by a series of phosphorylation events [98]. Next, APIs are transported outside the cell via specialized ATP-binding cassette transporters, interacting with transcription factors that control the expression of target genes [99]. A typical Gram-positive QS system consists of a membrane-bound histidine kinase receptor and a related cytoplasmic response regulator acting as a transcription regulator (Figure 2).

The most typical example of AIP-mediated QS is the *agr* system in *S. aureus* [92,95,99]. The *agr* system is evolutionarily conserved in Gram-positive bacteria, including *Lactobacillus plantarum*, *Clostridium botulinum*, *C. perfringens*, *C. difficile*, *L. monocytogenes* and *Enterococcus faecalis* [100]. In *S. aureus*, the synthesis of AIPs and their sensors are under the control of the P2 and P3 promoters, controlling the *agrBDCA* operon, which is transcribed to produce the polycistronic RNAII and RNA III transcripts [101,102]. The AIP precursor is encoded by the *agrD*, which, after subsequent modifications and the attachment of the thiolactone ring under the control of the *argB*, acquires the properties of a specific autoinducer API. The *agrC* is responsible for histidine kinase synthesis, while the AgrA, as a terminal regulatory protein, is synthesized under the control of the *agrA* gene [103]. *agrA*/*agrC* induces RNAII transcription, terminating the autoinduction and RNAIII circuits. Interestingly, instead of encoding a regulatory protein, the RNAIII transcript acts as a regulatory effector molecule for the *agr* system, mainly via translational inhibition of the virulence gene repressor Rot [103,104]. In *S. aureus*, a specific peptide sequence defines four groups of specific AIPs (I, II, III, IV) [105]. The *agrC*/*agrA S. aureus* system activates the expression of several virulence genes involved in α-hemolysin, coagulase, and enterotoxin synthesis [101,106]. A well-studied AIP system is ComQXPA *B. subtilis*, which comprises four proteins: the ComQ isoprenyl transferase, the ComX pre-peptide signal, the ComP histidine kinase, and the ComA response regulator [96,102]. ComQ is required to process, modify, export ComX, and produce the mature QS signal. Extracytoplasmic binding of ComX with ComP leads to phosphorylation and activation of ComA, which positively regulates surfactin production [107,108]. Another group of QS receptors is the RRNPP system, which was discovered in *Bacillus*, *Streptococcus*, and *Enterococcus* [96]. The RRNPP consists of Rap, NprR, PlcR, PrgX, and Rgg proteins [109]; the Rap is a phosphatase and transcriptional antiactivator, whereas NprR, PlcR, and PrgX are DNA-binding transcription factors. In *B. cereus*, NprR and PlcR regulate sporulation, virulence, biofilm formation, and genetic competence [96]. In *Streptococcus pyogenes*, Rgg regulates the expression of genes required for biofilm formation and virulence [110]. In turn, PrgX in *Enterococcus faecalis* regulates the conjugation of the antibiotic resistance plasmid pCF10 [111].

### 2.3. QS Based on Interspecies Communication

#### 2.3.1. Autoinducer System Type 2 (AI-2)

Autoinducer-2 (AI-2) is a conserved universal QS system coexisting in Gram-negative and Gram-positive bacteria [99,112,113]. The AI-2 system is believed to be used for cross-species signalling by organisms living in mixed-species communities, such as biofilms [99,114]. AI-2 produced by one species can influence gene expression in another, enabling bacteria to modify behaviours such as virulence, luminescence, and biofilm formation across different species [99,112,114]. For example, an EHEC strain that lacks the *luxI* gene can communicate within the species via Al-2 and sense Al-1 secreted by *P. aeruginosa* [113]. However, AI-2 produced by *E. coli* can be detected by *V. harveyi* to induce bioluminescence. Conversely, AI-2 produced by *V. harveyi* can be detected by *E. coli* to regulate the expression of the *lsr* system [114,115]. Moreover, AI-2 may coordinate microcolony formation and other processes in multispecies biofilms such as HGT [112,116].

The enzyme responsible for the synthesis of the AI-2 is the LuxS protein, a synthase encoded by the *luxS* gene [116]. LuxS is a metalloenzyme containing a zinc ion in the active site, which is involved in the cleavage of the ribose ring during the synthesis of AI-2 [117]. AI-2 is synthesized starting from S-adenosylmethionine, which through a series of enzymatic reactions, including the reaction catalysed by LuxS, is converted to 4,5-dihydroxy-2,3-pentanedione (DPD), a compound that cyclizes into several furanones in the presence of water [115]. DPD is a very reactive molecule that, in solution, spontaneously rearranges into a collection of chemically distinct molecular forms that contain AI-2 activity, which is recognised by receptor proteins of bacteria belonging to different species [118,119,120]. LuxS, the AI-2 has been identified in many bacterial species, including pathogens such as *E. coli*, *S. enterica* Typhimurium, *V. cholerae*, *Haemophilus influenzae*, *S. aureus*, *Streptococcus pyogenes*, *B. subtilis*, *C. jejuni*, *Helicobacter pylori*, *Klebsiella pneumoniae*, as well as *Shigella flexnerii* [118,121,122]. The LuxS/AI-2 QS system modulates various cellular processes involved mainly in the regulation of virulence factors, bacterial luminescence, sporulation, motility, toxin production, biofilm formation, and drug resistance [112,115,116,120].

In *Vibrio* species, AI-2 controls bioluminescence involving two proteins, LuxP and LuxQ [120,123]. AI-2/LuxP complex interacts with a sensor kinase, LuxQ, triggering a phosphotransfer cascade that leads to luciferase production and subsequent luminescence [99,120,121]. In *S. enterica* Typhimurium, the homologue of the LuxP is the LsrB (LuxS-regulated protein B) receptor, which is part of the ABC transporter system [124]. In this system, AI-2, by binding to the LsrB receptor, is phosphorylated by the LsrK kinase and, then, by binding to the transcription-regulating protein LsrR, activates the transcription of the *lsrACDBFGE* operon, resulting in active internalization of AI-2 from the extracellular space into the cytoplasm [119]. In pathogenic *H. pylori*, the function of the AI-2 is performed by the chemoreceptor TlpB, but the signal transduction mechanism has not yet been fully understood [117]. It is known, however, that AI-2 induces pathogenicity island genes in *E. coli* O157: H7 [125] and is involved in the regulation of hemolysin and protease synthesis in *V. vulnificus* [126], secretion of cysteine protease in *S. pyogenes* [127], and expression of the virulence gene *virB* in *Shigella flexnerii* [128]. In EHEC and enteropathogenic *E. coli*, LuxS is a crucial regulator of the QS and controls the expression of the T3SS system encoded by the locus of enterocyte effacement (LEE) pathogenicity island [46]. Transcriptomic studies have revealed that LuxS is a global regulator in EHEC, controlling the expression of over 400 genes [129]. Most of these genes have functions related to bacterial virulence, such as flagellar motility, surface adhesion, and Shiga toxin production [130].

#### 2.3.2. Autoinducer System Type 3 (AI-3)

The regulatory mechanism of the AI-3 autoinducer in biofilm formation and correlation with QS remains incomplete. In a previous study, the production of AI-3 was reported to depend on a *luxS* gene [46], but this was later shown to be due to an indirect effect [131]. It has been suggested that Al-3 may play an essential function as a QS signal in interspecies bacterial–host communication [132,133]. The AI-3 is a hormone-like signal transduced by the binary QseBC system in which QseC is a histidine kinase, whereas QseB is a response regulator [134]. The periplasmic QseC domain is preserved among several species of Gram-negative bacteria such as enteropathogenic *E. coli* (serotype O26: H11 and O111ac: H9), *Shigella* spp., *Salmonella* spp., *S. enterica* Typhimurium, *S. typhi*, *E. cloacae*, *Yersinia pestis*, *Y. enterocolitica*, *Pasteurella multocida* and *H. influenzae* [135]. AI-3 acts similarly to eukaryotic hormones since QseC is a bacterial adrenergic receptor for the eukaryotic host hormones epinephrine and noradrenaline [46,136]. Another consequence of this structural similarity is that AI-3 is inhibited by adrenergic receptor antagonists [135,137]. In addition, epinephrine/norepinephrine can provide a QS signal to the quorum of gut microbiota and activate the QseC/QseB system [137,138]. Enterohemorrhagic *E. coli* O157:H7 (EHEC) use human hormones such as epinephrine and noradrenaline to activate virulence genes [136,139], which can be associated with irritable bowel syndrome induced by chronic stress and the stress hormone cascade [132]. In *E. coli*, mobility and virulence are regulated by QS using an Al-3 signalling molecule [46,134,136]. In the presence of AI-3, the QseC domain undergoes autophosphorylation and then, by phosphorylating QseB, induces the transcription of the main flhDC regulon located in the locus of enterocyte effacement (LEE), which is responsible for cilia biosynthesis, cell mobility, and synthesis Shiga toxin [140]. However, the regulatory mechanisms of AI-3 for biofilm formation remain unclear.

#### 2.3.3. Bacterial–Host Communication

It is suggested that QS may control the species composition of the gut microbiota [114,141,142]. Thompson et al. [114] showed that antibiotic therapy’s disruption of the composition of gut bacteria species synthesizing AI-2 leads to dysbiosis. Interestingly, a much greater percentage of *Firmicutes* than *Bacteroidetes* encode functional AI-2 signalling systems [51,114]. It has been reported that AI-2 synthesized by gut microflora such as *Blautia obeum* was associated with reduced *V. cholerae* virulence and protection against this pathogen [143]. The human commensal bacterium *Ruminococcus obeum* was shown to inhibit colonization of the mouse gut by *V. cholerae*, partially through AI-2 signalling [143]. Moreover, AI-2 exposure to host epithelial cells has been associated with increased inflammatory cytokines, such as IL-8 [144] and IL-17A secretion, during acute *P. aeruginosa* infection [145]. In addition, Al-2 produced by *P. aeruginosa* caused apoptosis in some mammalian cells [141,146]. Recent studies suggest that QS is involved in bacterial–host interactions [141,142]. Ismail et al. [141] showed that mammalian epithelial cells produce an Al-2 mimic activity in response to bacteria or tight junction disruption that acts analogously to AI-2. This AI-2 mimic can be recognised by the bacterial AI-2 receptor, such as LuxP/LsrB, leading to the activation of QS-controlled gene expression [51,94,141]. AI-2 mimic could be involved in host–gut microbiota interaction and play a role in host–microbial symbiosis as epithelial cells directly interact with colonizing bacteria [141]. Although this remains debatable, AI-2 mimic may trigger widespread global gut microbiota gene expression changes.

The main bacterial QS systems used by selected bacteria are summarised in Table 1.

## 3. Molecular Mechanisms of the Formation and Functioning of Bacterial Biofilm

### The Role of QS in the Global Control of Gene Expression Profiles

Biofilm formation includes several stages, which depend on the colonized surface and the type of microorganisms [5,6]. The characteristic feature of bacterial cells that are an integral part of the biofilm is their increased resistance to external factors such as temperature, antibiotics, and nutrient changes [148]. These properties arise from the diversity of phenotypic subpopulations of bacterial cells forming the biofilm structure. Biofilm is characterised by complex ecological and structural heterogeneity, genetic diversity, the complexity of interactions, and the presence of extracellular substances [18,19,149,150]. The number of genes controlled by QS is large and may even exceed 10% of the bacterial genome [151,152]. Research on the molecular mechanisms of biofilm formation and the role of the QS in this process gained momentum with the development of high-throughput sequencing cDNA technology (RNA-seq) applying next-generation sequencing (NGS) platforms. Compared to the traditional methods of studying individual genes, transcriptomics provides a global study of gene expression and has been used successfully to study biofilm formation [129,153]. Numerous data revealed that pathogenic bacteria growing in biofilm exhibit differential gene expression (DEGs) compared with the planktonic state, including *Salmonella* [154], *S. pneumoniae* [155], *S. aureus* [156,157], *V. parahaemolyticus* [158], and *C. difficile* [159].

Generally, based on numerous transcriptional studies, the genes controlled by QS can be classified into four categories based on their biological functions [152,160,161]. The first group includes genes involved in cell life and growth; the second group includes genes controlling the behaviour of cells in the environment; the third group includes genes associated with HGT; and the fourth group includes genes whose expression is correlated with the synthesis of virulence factors [152,160]. Several groups of genes expressed by induction of the QS system, such as *Las* operon (*lasB*, *aprA*, *toxA*, *rhlR*), *Rhl* operon (*lecA*, *Lecb*, *rhlAB*), *Pqs* operon (*pqsE*), and *Igs* operon (*lasA*, *lasB*, *hcnA*, *rhlAB*), encode proteins belonging to proteases, elastases, coagulases, exotoxins, lectins, and other virulence factors [154,155,161]. Among the mRNA transcripts under the control of the QS system, different expression levels were noticed for genes involved in the stress response pathway (*hslS*, *hslT*, *soxS*) [156], as well as in the cellular metabolic pathway (*metK*, *artI*, *hyaA*, *fruK*, *gadB*) [154,156,157]. Recently, Jiang et al. [160] showed that the differential expression of *artM*, *artQ*, *ssrS*, *pflA*, and *hutX* genes (DEGs) was significantly correlated with the in vitro colonization and adhesion ability of *Haemophilus parasuis*; these are the most likely genes to affect biofilm formation. These data indicate that biofilm formation is a multifactorial process involving stress response, structural development, and regulatory processes. Nonetheless, it should be noted that some important signalling pathways can be regulated by phosphorylation cascades that are not detected at the level of the global expression analyses [152].

## 4. QS Pathways Inhibition

Quorum-sensing inhibitory compounds might be applicable in many fields, including medicine, agriculture, and environmental engineering. This is extremely important in the context of resistance to preventing and treating infections associated with the pathogenic biofilm resistant to traditional antibiotics. Many bacterial pathogens responsible for infectious diseases are known to have the ability to form biofilms. Due to the increased antibiotic resistance of human and animal pathogens, QQ is a promising antimicrobial approach. Prevention of biofilm formation by blocking the QS signal has the advantage that no direct bactericidal effect is associated with a lower probability of bacterial resistance development. In combination with antibiotic therapy, it increases its effectiveness by blocking the synthesis of a wide range of virulence factors [32].

**Figure 3:**
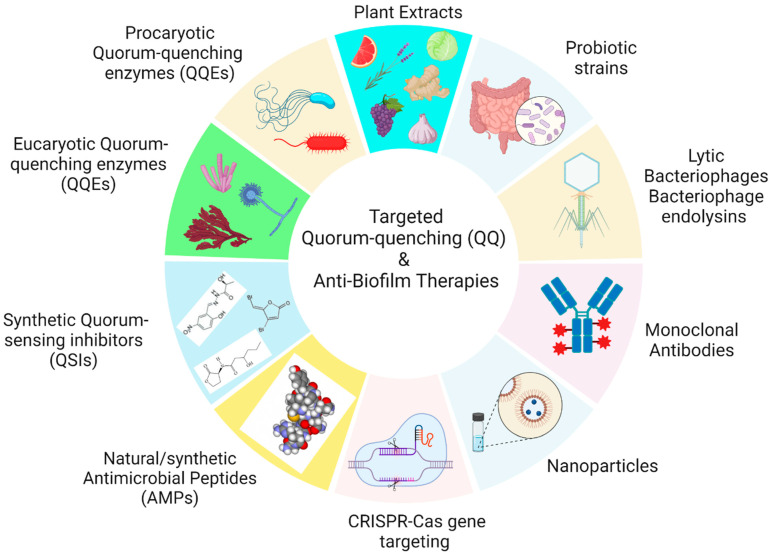
Schematic representation of possible mechanistic approach to reduce biofilm formation. The figure was created with BioRender (https://biorender.com/, 4 February 2023).

Therefore, next-generation antibiofilm agents are being discovered and developed to block particular virulence factors and specific matrix-targeting enzymes responsible for biofilm formation (Figure 3). There are different ways for QS inhibition in each pathway, such as (1) inhibition of AHL synthesis, (2) AHL receptor antagonism, (3) inhibition of targets downstream of receptor binding, (4) sequestration of AHL, (5) the degradation of AHL, and (6) inhibition of AHL secretion and/or transport [162].

### 4.1. Biotechnological Applications

#### 4.1.1. Natural and Synthetic QS Inhibitors

So far, many natural QS inhibitors have been isolated from bacteria, plants, fungi, and some animals from aqueous ecosystems [148]. These compounds are typically non-toxic to eukaryotes and offer many applications in medicine, food, and other industries. Natural compounds acting as QS inhibitors have been demonstrated in numerous species of herbs, vegetables, and fruits [163,164,165,166]. Furocoumarins, naturally occurring substances in grapefruit, showed more than 90% inhibition of the AI-1 and AI-2 activity in V. harveyi and biofilm formation by *E. coli* O157: H7, *P. aeruginosa*, and *S. Typhimurium* [164]. In *P. aeruginosa*, limonene extracted from mandarine (*Citrus reticulate*) inhibited biofilm formation by 41% at 0.1 mg/mL and AHL signalling production by 33%. Orange extract rich in flavons such as hesperidin, neohesperidin, and naringenin inhibited AHL production in *Yersinia enterocolitica* [165]. Antibiofilm activity was also observed for *Ananas comosus* extract (pineapple) or *Musa paradiciaca* (banana) water extracts, which prevented the synthesis of *P. aeruginosa* virulence factors such as proteases, elastases, and pyocyanin, which resulted in decreased biofilm production [167]. Murugan et al. [168] showed that the methanol extract from the herb *Andrographis paniculata*, containing diterpenoid lactone and andrographolide, effectively inhibited the production of bacterial efflux pumps and virulence factors in clinical strains of *P. aeruginosa* KMS P03 and KMS P05, resulting in increased sensitivity of bacteria to antibiotics and inhibition of biofilm formation [168]. Similarly, ethanol extract from *Amomum tsaoko* inhibited the biofilm formation of food-borne pathogens such as *S. typhimurium*, *S. aureus*, and *P. aeruginosa* [163]. In contrast, the biofilm formation of *E. coli* and *P. aeruginosa* was inhibited by the methanolic extract of *Buchanania lanzana Spreng* [169]. Pyocyanin production, biofilm formation, swarming motility, elastolytic, and proteolytic activities in *P. aeruginosa* PAO1 were inhibited by a flavonoid extract from *Centella Asiatica* [170]. *P. aeruginosa* PAO1 virulence was studied by Vandeputte et al. [166], who proved that specific flavonoids could decrease signal perception, which results in lower virulence and inhibition of biofilm formation. The ability of eugenol from clove, garlic, and phenolic extract of *Rubus rosifolius* to attenuate biofilm formation of *P. aeruginosa* and *Serratia marcescens* has also been reported [170,171]. Ruttrapa and Bais [172] showed that curcumin from *Curcuma longa* attenuated the virulence of *P. aeruginosa* PAO1 and prevented biofilm at the early stages of its formation. Recent studies have found that quercetin can inhibit the QS systems and target the *lasIR* and *rhlR* in *P. aeruginosa* and *lux* and *agr* in *Listeria monocytogenes*, respectively [173]. Kalia [164] showed antibiofilm QQ-dependent activity of secondary plant metabolites such as apigenin, naringenin, and kaempferol against *E. coli* O157:H7. Other plant extracts, such as hordenine and limonoids, have shown efficiency against biofilm formation by preventing the transcription of specific AHLs and were investigated as control strategies for inhibiting QS and biofilm formation [174].

Synthetic QQ molecules such as cinnamyl alcohol, allyl cinnamate, and methyl trans-cinnamate, which are derivatives of cinnamic acid, inhibited the production of the important virulence factor, violacein, by *Caenorhabditis violaceum* [175]. It has been reported that polyamine norspermidine effectively reduced the attachment of *P. aeruginosa* to the surface by inhibiting the expression of *lasI*, *lasR*, *rhlI*, *rhlR*, and *mvfR* genes [176]. Hobley et al. [177] showed that exogenous norspermidine prevented *B. subtilis* biofilm formation by condensing biofilm exopolysaccharide. Moreover, the class of chemically synthesized halogenated furanones has successfully inhibited biofilm formation [178,179]. Zhao et al. [178] reported that furanone C-30 may inhibit biofilm formation and antibiotic resistance in *P. aeruginosa* through regulating QS genes; significantly decreased *lasB*, *rhlA*, *phzA2*, *pqsR*, *lasI*, *rhlI*, *pqsE*, and *pqsH* expression levels in the mature biofilm have been observed. It was also shown that biofilms treated with C-30 are susceptible to tobramycin and readily dispersed by detergents [180]. In addition, the effect of C-5 aromatic substituted furanones on inhibiting biofilm formation and reducing virulence factor production in *P. aeruginosa* has also been reported [179]. Unfortunately, despite numerous advantages, recent reports indicate the development of bacterial resistance to QS inhibitors [152,164,181]. For example, studies of *mexR* and *nalC P. aeruginosa* mutants showed increased resistance to C-30 [181]. Defoirdt et al. [182] proposed that bacteria might evolve resistance to QQ compounds under conditions in which growth is directly coupled to QS. In addition, QS inhibitors can select more virulent strains, disrupting natural selection for reduced virulence [181]. Therefore, it is important to consider the risks associated with using the QQ strategies described above.

#### 4.1.2. Enzymatic QS Inhibitors

Enzymatic degradation of the QS signal is a second group of the QQ strategy. QQ enzymes were discovered in a wide range of bacteria and were classified into three major types according to their enzymatic mechanisms: (1) lactonase that hydrolyses lactone moiety of AHL; (2) acylase that cleaves amide bonds between lactone ring and the fatty acid side chain; and (3) oxidoreductase that modify AHL chemical structure by oxidation or reduction of a third carbon of the fatty acid side chain [183]. In Gram-negative bacteria, lactonase and acylase degrade all signals and have the broadest spectrum of AHL specificity regardless of acyl side chain length or substitutions [184]. AHL lactonases, such as SsoPox, Aii810, AiiK, AiiA, and AHL-1, isolated from different microorganisms, have been reported to sequester AHL and reduce biofilm formation [185,186,187]. Rajesh and Rai [188] showed that AiiA lactonase produced by the *Bacillus cereus* VT96 effectively inhibited biofilm formation and production of pyocyanin, rhamnolipid, and exopolysaccharides in *P. aeruginosa* PAO1. A reduction in lung injury and mortality in a rat *P. aeruginosa* model was also observed upon nasal administration of the SsoPox-1-lactonase, which inhibited QS signalling, virulence factor production, and biofilm formation [189]. Lactonase isolated from *Geobacillus kaustophilus* HTA426 was reported to degrade the lactone ring in the AHL’s structure, affecting Acinetobacter baumannii by impeding biofilm production [190]. Enzymes with lactonase activity, such as paraoxonases (PONs), have also been identified in host cells [191]. The ability of human PON1, PON2, and PON3 to AHL hydrolysis has been reported by Chun et al. [191]. Devarajan et al. [192] showed that in PON2 deficient mice, a marked impairment in their ability to hydrolyse 3-OC_12_-HSL and fight *P. aeruginosa* infection was observed. Similarly, in cystic fibrosis patients, lower PON-2 expression was associated with susceptibility to *P. aeruginosa* infection [193]. Gupta et al. [194] showed that lactonase obtained from *Bacillus* sp. ZA12 stopped the systemic spread of bacteria, reduced mortality, and offered synergistic activity with ciprofloxacin in a mice model of burn infection using the *P. aeruginosa* reference strain PAO1.

Acylase enzymes similar to lactonases can hydrolyse AHLs and disrupt the QS of pathogens bacteria. Acylases were derived from *Streptomyces* sp. M664 (AhlM) [195] *Ralstonia* sp. XJ12B (AiiD) [196], *Ralstonia solanacearum* GMI1000 (Aac) [197], *P. aeruginosa* (PydQ) [198], and *Ochrabactrum* sp. A44 (AiiO) [199]. In vitro experiments showed that AiiD and AhlM could greatly reduce the swimming of *P. aeruginosa*, extracellular elastase activity, secretion of pyocyanin, and the pathogenicity of nematodes [200]. Similar results have been reported by Utari et al. [198], who studied the activity of PvdQ on the AHL signalling molecule of *P. aeruginosa* in a mouse model. Results showed that PvdQ hydrolysed AHL, leading to a decrease in *P. aeruginosa* infection. Paul et al. [201] showed the potential of acylase I to reduce biofilm formation by *Aeromonas hydrophila* and *Pseudomonas putida* on borosilicate (36% and 23%), polystyrene (60% and 73%), and a reverse osmosis membrane. In the rabbit model of infection, the acylase, in combination with α-amylase derived from the *Bacillus amyloliquefaciens*, was found to degrade the biofilm formation of *E. coli* and *P. aeruginosa* [202]. In turn, *Aspergillus melleus* acylase incorporated within silicon catheters and polyurethane coatings disrupted the biofilm formation of *P. aeruginosa* ATCC10145 and PAO1 strain [202].

Regarding oxidoreductases, the novel oxidoreductase BpiB09 derived from the metagenomic library was found to be able to inhibit 3OC_12_-HSL production, leading to a significant reduction of motility, biofilm formation, and pyocyanin synthesis in *P. aeruginosa* [200]. The P-450/NADPH-P450 isolated from *B. megaterium* CYP102A1 was capable of the efficient oxidation of AHLs at the ω-1, ω-2, and ω-3 carbons of the acyl chain to eliminate their QS activity [203]. Uroz et al. [204] reported the presence of two oxidoreductases in *Rhodococcus *erythropolis** W2, which converts 3-oxo-AHLs to their corresponding 3-hydroxy derivatives, and an amidolytic activity, which cleaves the amide bond linking the acyl chain to the HSL residue. Similarly, the capability of QQ-2 oxidoreductase, immobilized to the glass surface, to inhibit *Klebsiella oxytoca* and clinical *K. pneumoniae* biofilm formation, has also been reported [205].

#### 4.1.3. Antimicrobial Peptides as QS Inhibitors

Antimicrobial peptides (AMPs) are a class of natural (NAMPs) and synthetic peptides (SAMPs) with a broad spectrum of antimicrobial properties [36,206]. Natural AMPs are important components of the innate immunity of almost all living organisms, protecting the host against infections [206,207]. NAMPs have been extracted from bacteria, fungi, plants, insects, fish, amphibians, mammals, and the human body [206]. The largest number of AMPs derived from animals, totalling 2519 AMPs, followed by 824 AMPs from plants, 431 AMPs from bacteria, 7 AMPs from protozoans, 6 AMPs from fungi, and, finally, 4 AMPs from archaea [208,209]. In various studies, AMPs have exhibited antibacterial and antibiofilm activity against various MDR strains and, therefore, are promising alternatives to current antimicrobials [36,206,207]. Antimicrobial properties of NAMPs, including gramicidin S from *B. brevis* [210], polymyxin B and A from *B. polymyxa* or vancomycin produced by *S. orientalis* [211], have been reported in several studies [207,212]. Similarly, magainin-2 extracted from amphibians, such as frog skin, showed antibacterial activity against MDR strains, protozoa, yeasts, and fungi [213]. Crotalicidin extracted from rattlesnakes killed 90% of *E. coli* and *P. aeruginosa* cells within 90–120 min and 5–30 min, respectively [214]. Moreover, the strong in vitro antibacterial potential of NAMPs against various pathogenic microorganisms isolated from marine sources has also been reported [209]. Polyphemusin-I obtained from hemocyte debris of *Lumulus polyphemus* showed antibacterial activity against *E. coli* and *Candida albicans* [215]. Raghavan et al. [216] reported that MFAP9 derived from marine *Aspergillus fumigatus* BTMF9 exhibited inhibitory activity against *B. circulans* biofilm formation. Cathelicidins (CATH BRALE and codCath1) derived from fish showed antibacterial activity in a broad spectrum of Gram-positive and Gram-negative bacteria [175]. The best-studied NAMP produced in the human body is cathelicidin LL-37, termed host defence enzymes, which possesses antimicrobial and antibiofilm activities against a broad spectrum of MDR strains [171,217]. A large number of studies regarding antimicrobial/antibiofilm properties of the LL-37 are focused on strains in which antibiotic resistance is a serious problem, including *P. aeruginosa* [218], *S. aureus* [219], *S. epidermidis* [220], *Streptococcus pneumoniae* [221], *Streptococcus pyogenes* [222], *Acinetobacter baumannii* [223], *E. coli* [224], *K. pneumonia* [225], *Helicobacter pylori* [226], and *Aggregatibacter actinomycetemcomitans* [227]. In *P. aeruginosa* PAO1 grown under biofilm conditions in a flow cell, global gene expression analysis revealed that 4-day exposure to LL-37 (4 µg/mL) led to the downregulation of 475 genes, including QS-controlled genes such as *lasl* and *rhlR* [228]. This caused the downregulation of over 50 genes that are part of the respective regulons and affected the transcription of genes involved in producing virulence factors, motility, adhesion, the development of biofilm, and the modulation of host immune responses [189]. Xiao et al. [218] showed that sub-growth inhibitory doses of LL-37 affect biofilm formation in *P. aeruginosa* PAO1 by reducing the elastase and pyocyanin levels, promoting eDNA release and biofilm formation. In addition, LL-37 at a concentration of >20 µM suppressed *S. aureus* biofilm formation, isolated from lesion skin of patients with atopic dermatitis [229]. In addition, LL-37 reduced biofilm formed by MRSA at 41% [230]. Tachyplesin III from Southeast Asian horseshoe crabs is also known for its antimicrobial properties [231]. Minardi et al. [231] showed that Tachyplesin III, in combination with piperacillin-tazobactam, significantly reduced *P. aeruginosa* biofilms in a rat ureteral stent model. Moreover, antibiofilm properties of Protegrin 1 against *Acinetobacter baumannii* [232], indolicidin against multi-drug-resistant enteroaggregative *E. coli* (MDR-EAEC) [233], as well as SMAP-29 against *Burkholderia thailandensis* isolated from pig [232], cattle [234], and sheep [235], have also been demonstrated.

AMPs affect biofilm formation or degradation with different mechanisms of action, including acting on the cell wall, cell membrane, and different intracellular targets, as well as host immune system modulation activities [236]. Some AMPs destroy bacterial cell wall structure by interfering with the biosynthesis of cell wall components such as peptidoglycan [237,238]. Vancomycin and oritavancin can bind to the cell wall synthesis precursor lipid II, which in turn interferes with further enzymatic processes, thereby inhibiting peptidoglycan synthesis [237]. Similarly, nisin secreted by *Lactococcus* and *Streptococcus* exerts an antibacterial effect by inhibition of peptidoglycan synthesis and forms pores at sensitive membranes upon interaction with lipid II synthesis [238,239,240]. Moreover, peptides can inhibit cell wall and protein synthesis, bacterial cell division or DNA replication by interacting with specific proteins involved in this biological process. Di Somma et al. [241] showed that temporin-L interaction with *E. coli* FtsZ protein impaired cell division by inhibiting Z-ring formation, causing bacterial death without damaging the cell membrane. Mardirossian et al. [242] showed the antimicrobial activity of Bac5 against *E. coli*, *A. baumannii*, *K. pneumoniae*, *S. aureus*, *S. enterica*, and *P. aeruginosa* by inhibiting bacterial protein synthesis. A similar antibiofilm mechanism for proline-rich AMPs [243] and several SAMPs, e.g., PS1-2, 35409 or SET-M33 [209,234,236], has also been demonstrated. Moreover, studies reported that SAMPs are more efficient NAMPs by exerting antibacterial activity at low concentrations than their natural analogues [243,244]. For example, compared to natural AamAP1, synthetic AamAP1-Lysine had stronger antibacterial activity and bactericidal efficacy against *S. aureus* and *E. coli* in the low concentration range of 5–7.5 µM [244].

Although large numbers of AMPs have been characterised, a small number have been applied in clinical trials, and a limited number have been approved by the US Food and Drug Administration (FDA) [245]. Most clinically used AMPs are limited to topical applications due to their systemic toxicity, the susceptibility of the peptides to degradation by proteases, and rapid kidney clearance when administrated orally [246]. Furthermore, oral administration of AMPs can lead to proteolytic digestion by digestive enzymes, such as trypsin and pepsin, while systemic administration leads to a short half-life, protease degradation, and cytotoxic profiles in blood [246].

#### 4.1.4. Antibodies for Quenching QS Signalling

In vitro and in vivo studies have reported the effectiveness of monoclonal antibodies (mAb) against QS signal molecules and biofilm formation, especially bacterial pathogens [247,248,249]. Antibodies acting against AI molecules could disrupt cell-to-cell and cell–surface interactions, thereby interfering with biofilm formation [248,250]. Although many antibacterial mAbs are still under experimental investigations, the QQ antibodies represent a promising treatment strategy that may complement antibiotic therapy to improve treatment for biofilm-associated infections [250]. In the pioneering study from Kaufmann et al. [251], murine anti-AHL antibody RS2-1G9 inhibited QS signalling and QS-regulated pyocyanin in vitro production in *P. aeruginosa* via binding 3OC_12_-HSL. The MAb RS2-1G9 was also tested for its ability to protect murine macrophages from the cytotoxicity effects of the *P. aeruginosa* quorum sensing molecule 3-OC_12_-HSL, and it was demonstrated that RS2-1G9 protected macrophages from v-induced apoptosis. The antibody also prevents the activation of cellular stress kinase pathways, indicating that the sequestration of 3-OC_12_-HSL is complete [252]. In the study from Sun, Accavitti, and Bryers [253], three isolated mAbs, namely 12C6, 12A1, and 3C1, against *S. epidermidis* cell wall accumulation-associated protein (AAP) inhibited biofilm formation on abiotic surfaces. Moreover, significantly higher biofilm inhibition was noticed for mAb mixtures compared with individual mAb. The ability of biofilm inhibition by 12C6, 12A1, and 3C1 was 42%, 39%, and 66%, respectively. However, 12A1 and 3C1 mixtures and 12C6 and 12A1 increased *S. epidermidis* RP62A biofilm formation inhibition to 87% and 79%, respectively. In turn, a human mAb, TRL068, was shown to disrupt *S. aureus* and *S. aeruginosa* biofilm formation via binding to the DNABII proteins, resulting in the rapid collapse and subsequent detachment of bacteria from their protective biofilm matrix. This leads to the subsequent pathogen clearance by host immune effectors or antibiotics [254]. In addition, TRL068 showed the effectiveness of in vitro biofilm inhibition of *E. faecium*, *S. aureus*, *K. pneumoniae*, *A. baumannii*, *P. aeruginosa*, and *Enterobacter* spp. (ESKAPE) pathogens. Moreover, antibiofilm activity of TRL068 has also been reported in experimental biofilm models of chronic human diseases, including otitis media (OM), caused by nontypeable *Haemophilus influenzae* (NTHi) in chinchillas, lung infection by *P. aeruginosa* in mice, and periodontal peri-implantitis by *Aggregatibacter actinomycetemcomitans* in rats [255,256]. Park et al. [195] reported that mAb AP4-24H11 against the *agr* locus efficiently inhibited QS in vitro via sequestration of the autoinducing peptide AIP IV produced by *S. aureus* RN4850 and reduced the α-hemolysin expression. Moreover, an in vivo study has demonstrated that mAb AP4-24H11 significantly attenuated the pathogenicity of *S. aureus* in the infected mouse model [195,257]. In addition, antibody-based QQ also involved other strategies, such as generating catalytic antibodies to degrade and thus inactivate the AHLs. De Lamo Marin et al. [258] used this approach to screen and evaluate catalytic antibodies for lactonase activity. A mAb XYD-11G2 was shown to suppress pyocyanin production by hydrolysing 3OC_12_-HSL in *P. aeruginosa* cultures. Several human mAbs capable of binding biofilm and planktonic forms of S. aureus, including 4497-IgG1, CR5132, and rF1-IgG1, have recently been identified [259]. De Vor et al. [259] demonstrated that these antibodies had a great ability to block *S. aureus* biofilm formation via direct binding to wall teichoic acid (WTA) or surface proteins of the serine–aspartate dipeptide repeats (SDR) family.

Although monoclonal antibodies effectively block QS signalling among pathogenic bacterial species, their applications for treating bacterial infections are still in the initial stage [260]. Several antibodies, including ClfA, CP5 and 8, PNAG, Hla, and HlgAB targeting *S. aureus* biofilm, have been tested as passive vaccines in clinical phase II and/or III trials [261,262,263]. However, none of them improved the clinical outcome in treating bacteremia and cystic fibrosis patients [261,262,263,264]. Nevertheless, several interesting *S. aureus* vaccine candidates have shown promising results in pre-clinical studies [265,266]. For instance, MEDI3902 against *P. aeruginosa* biofilm formation received a fast-track designation from the FDA in 2014 [265]. Currently, several other mAbs targeting *S. aureus* toxins and immune evasion proteins, e.g., ASN-100 (Arsanis) and 514G3 (X-Biotech), are being tested in different phases of clinical trials [266].

#### 4.1.5. Nanoparticles Strategy of QS Inhibition

Blocking the activity of the QS system with metal or metal-oxide nanoparticles (NPs) is a new strategy in the fight against pathogenic microbes [267,268,269,270]. Due to the strong antimicrobial properties of NPs, their pleiotropic effect on the cell, non-toxic, relatively safe, and specificity towards QS systems, they are gaining increasing importance in treating bacterial infections [268]. Most research on their therapeutic function concerns mainly *P. aeruginosa* [271], *S. aureus* [272], and *E. coli* [273,274]. NPs based on silver (Ag NPs), gold (Au NPs) or zinc oxide (ZnO NPs) are effective QQ due to their ability to inhibit bacterial microcolony formation, reduce biofilm production, and change its structure [275]. The antibiofilm activity of Ag NPs has been demonstrated in numerous studies [276,277] and summarised in comprehensive reviews [278,279]. Ag NPs are highly effective against *P. aeruginosa* and inhibit the transcription of the *phzA-G* operon and piochelin, pyoveridin, and rhamnolipids synthesis [271,280]. In biofilm-forming *P. aeruginosa*, Ag NPs disrupt proteins due to the binding of ionic constituents to cysteine residues, causing more deterioration and impairing the formation of exo-polysaccharides [280]. The antimicrobial activity of Ag NPs against planktonic forms of *E. coli* and the inhibition of biofilm formation has been reported by Du et al. [281]; the Ag NPs reduced *E. coli* biofilm formation in vitro by inhibiting bacterial adhesion and *icaAD* expression. On the other hand, Yang et al. [282] reported that the antibacterial activity of Ag NPs is more effective against Gram-negative (*E. coli*) than against Gram-positive bacteria (*S. aureus* and *S. epidermidis*) and yeast (*Candida albicans*). Starch-stabilised Ag NPs have been found to inhibit biofilm formation by food-borne pathogens like *Shigella flexneri*, *Salmonella typhi*, and *Mycobacterium smegmatis* and are non-toxic to macrophages. In addition, these Ag NPs were more potent as antibiofilm agents than antimicrobial peptides, such as LL-37 [283]. In addition, Au NPs have been shown to exhibit strong antibiofilm activity against *P. aeruginosa* PAO1 by reducing exo-polysaccharides synthesis [284].

Recently, there has been increased interest in zinc oxide nanoparticles (ZnO NPs). This is mainly because ZnO is one of the metal oxides listed as Generally Recognized As Safe (GRAS) by the FDA due to its non-toxic properties. [269,283,285]. Numerous studies have been reported on ZnO NPs’ efficiency in inhibiting broad-spectrum pathogens’ growth [273,280,285,286], which could potentially replace conventional antibiotics. Kemung et al. [273] reported that the anti-adherence and antibiofilm properties of ZnO NPs against MRSA *S. aureus* were higher than the antibiotic vancomycin, even at low concentrations. Moreover, evidence has indicated that ZnO NPs exhibit potential applications in the poultry and livestock industries, particularly as a feed supplement in the animal’s diet [285]. Antibacterial and antibiofilm properties of ZnO NPs against *P. aeruginosa* PAO1, *E. coli* O157:H7 (EHEC), methicillin-resistant *S. aureus* (MRSA), and a methicillin-sensitive *S. aureus* (MSSA) have been reported by Lee et al. [280]. However, Khan et al. [285] showed that ZnO NPs effectively inhibited the biofilm formation of oral opportunistic pathogens, *Rothia dentocariosa*, and *Rothia mucilaginosa.* Another study demonstrated the antibiofilm activity of ZnO NPs against food-borne pathogens such as *S. aureus*, *S. enterica*, and *E. coli* [274]. Furthermore, Vinotha et al. [286] reported that synthesized ZnO NPs using an insulin-rich leaf from *Costus igneus* showed antibiofilm activity against *Streptococcus mutans*, *Lysinibacillus fusiformis*, *Proteus Vulgaris*, and *Vibrio parahaemolyticus*.

An antibiofilm effect has also been observed for CuO NPs, effectively destroying biofilm produced by MRSA *S. aureus* strains and *E. coli*. In *Methylobacterium* spp., CuO NPs coupled with carbon nanomaterials inhibited QS and prevented biofilm formation [287,288]. Moreover, the antimicrobial and antibiofilm capabilities of MgO and aluminum oxide (Al_2_O_3_) NPs on planktonic and biofilm forms of antibiotic-resistant *E. coli*, *K. pneumoniae*, and *S. aureus* have also been demonstrated [289,290].

Recent studies suggest that bacteria can develop resistance to NPs after long-term exposure [291,292,293]. Kaweeteerawat et al. [291] showed that Ag NPs can enhance bacterial resistance to antibiotics by promoting stress tolerance via the induction of intracellular ROS. Panáček et al. [292] showed that *E. coli 013*, *P. aeruginosa* CCM 3955, and *E.coli* CCM 3954 can develop resistance to Ag NPs after repeated exposure to increased production of the adhesive flagellum protein flagellin, which stimulates the aggregation of Ag NPs and destruction their antibacterial effect. Additionally, in several studies, toxic effects of the same NPs have been reported [245,293]. For example, Hemeg [294] showed that Ag NPs can accumulate in human organs like the colon, liver, spleen or bone, causing damage and/or decreased organ efficacy and dysfunction. In turn, exposure to Al_2_O_3_-NPs may produce reactive oxygen species (ROS) within the cells and impair the level of antioxidant activities [295]. Ji et al. [296] demonstrated that intranasal instillation of Al_2_O_3_ NPs led to oxidative damage in the brains of ICR mice, impaired neurobehavioural functions, and induced cell necrosis and apoptosis ROS production and oxidative damage induced by CuO NPs and ZnO NPs has also been reported [294]. Therefore, further studies are needed to verify the potential development of bacterial resistance to NP exposure.

#### 4.1.6. Probiotic Therapies Based on QS Inhibition

Due to the abundance of commonly used antibiotics in recent decades, antibiotic resistance of pathogen strains is ubiquitous and difficult to control. Gut microflora dysbiosis is associated with various human diseases, including type 2 diabetes, cardiovascular disease, Clostridium difficile infection (CDI), colorectal cancer, and obesity [297,298]. By adopting *S. typhimurium*, Enterohaemorrhagic *E. coli* (EHEC), and *Clostridium difficile* as representative pathogens, Bäumler [299] conducted comprehensive studies based on the interactions between the gut microbiota, the host, and the above-mentioned pathogens and antibiotic therapy. The study has shown that antibiotic treatment increased the level of free sialic acid (from the host) and succinate (from the gut microbiota), which in turn promoted the expansion of *Salmonella typhimurium* and *Clostridium difficile* and damaged the intestinal epithelial cells. In addition, EHEC has been found to use a QS system with fucose sensors to avoid nutrient competition with commensal *E. coli* [300]. To reduce the defect of antibiotic treatments that cause resistance to pathogenic bacteria, many attempts have been made to develop probiotic therapies based on lactic acid bacteria (LAB) as vectors for drugs and signalling molecules [301]. Moreover, probiotic delivery techniques not only inhibited the biofilm formation of pathogenic bacteria but also stimulated the host immune system [302]. Studies have shown that certain probiotic strains may interfere with the QS system of ESKAPE bacteria, inhibiting pathogenic biofilm from its initial stage of attachment and development to its dispersion [42,187]. Valdez et al. [303] demonstrated that *Lactobacillus plantarum* PA100 can prevent the induction of *P. aeruginosa* virulence factors by targeting AHL. According to this study, the development of biofilm, elastase, and AHL could be inhibited by the acid filtrate and the neutralized filtrate of *L. plantarum* PA100. In addition, the effect of *L. crustorum* ZHG 2-1 (*Companilactobacillus crustorum*) on the suppression of C4-HSL and 3-oxo-C12-HSL synthesis leading to the inhibition of *P. aeruginosa* biofilm formation and reduction of virulence factors (chitinases and proteases) was also noticed [130]. Chapman et al. [304] showed that multi-strain probiotic preparation of *L. acidophilus* NCIMB 30184, *L. fermentus* NCIMB 30226, *L. plantarum* NCIMB 30187, and *L. rhamnosus* NCIMB 30,188 inhibited biofilm formation of pathogenic bacteria such as *Clostridium difficile*, *E. coli*, and *S. Typhimurium*. The ability of *L. brevis* to inhibit pyocyanin production and biofilm formation in *P. aeruginosa* strain PA002 has been demonstrated by Liang et al. [305]. Moreover, the metabolites of LAB (*L. lactis* NCDC 309, *L. rhamnosus* MTCC 5897, *L. rhamnosus* MTCC 5857, *L. fermentum* MTCC 5898, *L. acidophilus* NCDC 15, *L. delbrueckii* subsp. *lactis*, and *L. plantarum* NCDC 372) were found to effectively inhibited elastase and biofilm formation, as well as *lasI* and *rhlI* expression in *P. aeruginosa* [306]. QS in *Listeria monocytogenes* was inhibited by the metabolites of *L. plantarum* M.2 and *L. curvatus* B.67 due to inhibition of *agr* genes [307]. A similar mechanism has been noted for *C. difficile*, which has been shown to inhibit AI-2 and the *luxS* system upon adding heat-treated supernatant *L. fermentum* Lim2 [308]. Furthermore, lipopeptides known as phengycins produced by *Bacillus subtilis* have been shown to interfere with the QS system of *S. aureus* by suppressing *agr* signal transduction, leading to inhibition of the production of key Agr-regulated virulence factors such as phenol-soluble modulins, α-toxin, and Panton–Valentine leucocidin [309]. Similar to the previous example, the biosurfactants generated by *L. plantarum* and *Pediococcus acidilactici* decreased the expression of AI-2 in a dose-dependent manner, as well as the *cidA*, *icaA*, *dltB*, *agrA*, *sortaseA*, and *sarA* genes, which are related to biofilm formation in *S. aureus* [187]. In addition, the effectiveness of other probiotic strains such as *L. reuteri* RC-14 [310], *Bifidobacterium* BB12 [311], and *Bifidobacterium adolescentis* SPM1005 [312] in QS system suppression and inhibition of the pathogenic biofilm formation has also been reported.

#### 4.1.7. Bacteriophage Application

In recent years, bacteriophages (phages) have re-gained interest mainly due to their host specificity and bacteriolytic activity against antibiotic-resistant strains and their biofilms [313,314,315]. Applying phages in bacterial biofilm eradication involves using naturally occurring strictly virulent or lytic phages that do not encode genes for virulence, toxins or AMR [313,315]. Phage should not be able to mediate horizontal gene transfer or transduce infected bacterial cells [316]. Single phages usually have a narrow host range as they are generally specific for a limited set of strains of the same bacterial species [316]. A phage mixture or cocktail is commonly used to target either mono or several bacterial strains due to its greater efficacy in biofilm destruction than a single phage application [317,318,319]. The use of phage cocktails arises from the fact that simultaneous treatment targeting a variety of bacterial receptors with diverse antibacterial pathways will more efficiently decrease the bacterial burden, expand host range coverage and lysis potential, and mitigate resistance or development of lysogenic strains [316,320].

In numerous in vitro biofilm studies, phages have shown their efficacy in penetrating established biofilms and eradicating bacteria [321], and the effectiveness of single phages and phage cocktails to infect and lyse bacterial cells in single and multispecies biofilms has been confirmed [314,315,316,322]. Recent reports found that phages are highly effective at in vitro reducing and controlling bacterial biofilms, particularly those formed by *S. aureus*, *K. pneumoniae*, *Acinetobacter baumannii*, *P. aeruginosa*, *Listeria monocytogenes*, *Salmonella* sp., and *E. coli* [40,323,324,325,326]. For example, Peng et al. [327] demonstrated that phage ɸMR003 displayed a broad host range against methicillin-resistant *S. aureus* of human origin. Kazimierczak et al. [328] demonstrated that phages vB_SauM-A, vB_SauM-C, and vB_SauM-D were effective against most multi-drug-resistant *S aureus* strains and, additionally, showed more efficiency in biofilm reduction compared to the antibiotics used. Moreover, antibiofilm properties of other isolated phages, such as vB KleM-RaK2 (RaK2) against *Klebsiella* sp. [329], phiPA3 against *Pseudomonas aeruginosa* [330], phiRSL1 against *Ralstonia* sp. [331], vB_EcoM_10C2 and vB_EcoM_11B2 against E. coli O177 [332], and BPECO 19 against *Escherichia coli* O157:H7 [333], as well as R1-37 against *Yersinia enterocolitica* [334], have been determined. Several studies report the success of lytic phages against enterococci biofilms. Melo et al. [322], for instance, showed that newly isolated phages, the siphovirus y BEfaS-Zip (Zip) and the podovirus vB EfaP-Max (Max), demonstrated lytic activity against *E. faecalis* and *E. faecium*, which are the most frequent antibiotic-resistant strains present in chronic wounds. Rakov et al. [335] showed that phages PSTCR4 and PSTCR6 exhibited an efficient reduction of well-established MDR *Providencia stuartii* biofilm formed in the catheter model. D’Andrea et al. [336] reported that vB_EfaH_EF1TV phage belonging to the *Herelleviridae* family inhibited biofilm produced in vitro by *E. faecalis* clinical strains. In a study by Khalifa et al. [337], phage EFDG showed effective lytic activity against various antibiotic-resistant *E. faecalis* and *E. faecium* isolates and disrupted their biofilms. However, Bhardwaj et al. [338] found a phage targeting multi-drug-resistant *Enterococcus* strains isolated from chronic periodontitis patients, and its ability to reduce biofilm formation by *E. faecalis* after 24 h of infection was observed.

Recent studies showed that applying phage cocktails in biofilm models is highly efficient at destroying bacterial biofilms [313,314,315]. For example, in vitro lytic efficacies of phage cocktails AB-SA01 and AB-PA01, which target *S. aureus* and *P. aeruginosa*, respectively, significantly reduced biofilm biomass in mixed-species biofilms, compared to the respective phage cocktail treatment [339]. Gutierrez et al. [340] demonstrated that the mixture of phiIPLA-RODI and phiIPLA-C1C phages was more efficient in the planktonic phase than in the biofilms phase during *S. aureus* IPLA16 and *S. epidermidis* LO5081 mixed-species cultures. Similarly, phages ΦKpnM-vB1, ΦKpnP-vB2, and ΦKpnM-vB3 were highly efficient in reducing *K. pneumoniae* biofilms when applied as a cocktail [341]. Similarly, the phage cocktail composed of four lytic ΦEcp1, ΦEcp2, ΦEcp3, and ΦEcp4 phages completely inhibited the growth of MDR *E. coli* and significantly prevented the development of biofilms. The phage mixture caused strong biomass reduction of biofilm and showed the highest biofilm inhibition, up to nearly 87% [318]. Several experiments had more extensive bactericidal results when phage therapy was combined with antibiotics as a single treatment [328,342]. Jiang et al. [342] showed that virulent phage WV in high-concentration *S. aureus* culture demonstrated a greater antibiofilm effect than streptomycin. In addition, using phage WV and streptomycin in combination yielded significantly better antibiofilm and bactericidal effects against *S. aureus* than those achieved using streptomycin or phage WV alone [342].

Recent advances in biotechnology and synthetic biology fields have enabled the development of various methods of phage genetic engineering to modify their host range and improve safety and antimicrobial activity [343,344,345]. Several engineering phages to express degradation enzymes targeted at the EPS matrix for biofilm destruction have been reported [346,347,348]. For example, the modified T7 phage with expressed dispersin B enzyme effectively reduced more than 99% of *E. coli* biofilm [349]. Additionally, T7 phage expressing AiiA lactonase was reported to effectively reduce the QS of *P. aeruginosa* in a mixed E. coli biofilm, resulting in a 75% and 66% reduction in biomass after 4 and 8 h, respectively [347]. Møller-Olsen et al. [350] used CRISPR-Cas-based selection to obtain a T7-like phage, K1F, which was able to kill inside human cells a hybrid between *E. coli* strains K12 and K1, responsible for urinary tract infections, meningitis, and sepsis. More recently, the first clinical application of an engineered phages cocktail (Muddy, ZoeJ, and BPs) was applied to treat a cystic fibrosis patient with a disseminated *Mycobacterium abscessus* infection [351].

It is important to note that a fundamental principle of phage therapeutic development for clinical purposes is to ensure the potential phage product is safe and effective. Despite all the successful cases of patients treated with phages documented to date [352,353,354,355,356], the introduction of phage therapy in Western countries still faces major barriers, especially regulatory issues [357]. The main limitation of phage therapy is high host specificity and the possibility of developing resistance by targeted bacteria against phage attachment and adsorption by altering the receptor sites [245]. Additionally, it is difficult to control the stability and purity of phages that are prepared for clinical trials, which may result in low-quality control data [358]. Moreover, a significant decrease in phage concentrations by the reticuloendothelial system or neutralization by antibodies during therapeutic application has also been reported [359].

Now, attempts to make phage therapy widely available are underway, and several clinical trials are being carried out in Europe and the United States (US) [360,361]. For example, a clinical trial including a phase 1b/2 trial assessing the microbiological activity of a single dose of phage therapy in cystic fibrosis patients chronically colonized with *P. aeruginosa* is conducted by the APT, Inc., with Antibacterial Resistance Leadership Group (ARLG) cooperation (https://aphage.com/adaptive-phage-therapeutics-announces-first-patient-dosed-in-the-phage-clinical-trial/, 23 January 2023). Additionally, in 2022, Locus Biosciences, Inc., kicked off a randomized phase 2/3 trial evaluating the safety, tolerability, pharmacokinetics and efficacy of a CRISPR-enhanced phage (crPhage^®^) for the treatment of urinary tract infections (UTIs) caused by MDR *E. coli* bacteria (https://www.locus-bio.com/locus-biosciences-announces-first-patient-treated-in-the-eliminate-registrational-phase-2-3-trial-of-lbp-ec01-for-urinary-tract-infections/, 13 September 2022). On the other hand, the application phage preparations in the agro-food sector have already been approved and supported by authorities in certain countries, such as the US, where biopreparations against *Listeria monocytogenes* (Listshield^TM^), *S. enterica* (SalmoFresh^TM^), and *E. coli* (Ecoshield^TM^) for direct application to food are commercially available [362]. QQ mechanisms of antimicrobial/antibiofilm activity are summarized in Table 2.

### 4.2. Genome Applications

#### 4.2.1. Therapies Based on the CRISPR/Cas Systems

Palindromic repeat–CRISPR-associated (CRISPR/Cas) systems have been identified as a bacterial adaptive immune system [394,395] and found in approximately 50% of bacterial genomes and 87% of archaeal genomes [396,397]. The genetic loci of CRISPR/Cas systems contain the CRISPR array, comprising short repeated sequences (repeats) and similarly sized flanking sequences (spacers). The Cas proteins encoded by *cas* genes, located in the proximity of a CRISPR array, are key functional elements of CRISPR systems that offer adaptive immune protection against bacteriophages or other foreign mobile genetic elements [398]. In bacteria, CRISPR/Cas systems, according to the diversity of *cas* genes, are categorized into 2 classes, 6 types (I-VI), and 33 subtypes [395,399]. Each CRISPR/Cas system has a specific protein composition for expression, interference, and adaptation [394,395,400]. Class 1 comprises three types (I, III, and IV) and sixteen subtypes, whereas Class 2 includes three types (II, V, and VI) and seventeen subtypes [401,402]. The Class 1 CRISPR/Cas system takes on interference through the use of a multi-Cas effector protein complex, whereas Class 2 utilises a single effector protein responsible for the identification and cleavage of the target sequence [403]. Among the type II CRISPR/Cas systems, the most commonly studied effector protein is the DNA endonuclease Cas9 using a specificity-programming guide RNA (gRNA). The gRNA is a specific RNA sequence that recognises the target DNA region of interest and directs the Cas9 for editing [398,399,403]. Currently, Cas9 isolated from *Streptococcus pyogenes* (SpCas9) is extensively carried out for gene edition due to its simplicity, versatility, efficiency, and specificity [396,400,403].

In recent years, the CRISPR/Cas9 system has emerged as a promising tool for developing next-generation antimicrobial agents to combat infectious diseases, especially those caused by AMR pathogens [395,403]. CRISPR/Cas9 has been widely applied in targeting genes that encode antibiotic resistance and virulence in bacteria [404]. Depending on the localisation of the target gene, CRISPR/Cas9 can be used in two different ways, a pathogen-focused approach and a gene-focused approach [405,406]. A pathogen-focused way is targeting specific chromosome regions to induce bacterial cell death. On the other hand, targeting the plasmids that carry the AMR genes is part of the gene-focused approach. This way removes the plasmid and causes bacteria to be susceptible to antibiotics [407,408].

In several studies, CRISPR/Cas9 has been successfully used to selectively remove target genes involved in antibiotic resistance of clinical pathogens [408,409]. For example, Bikard et al. [410] used the CRISPR/Cas9 system to target the *mecA* gene conferring methicillin resistance to clinical isolate *S. aureus* USA300, which significantly reduced the *S. aureus* counts (50%) from a mixed population of bacteria as compared to the control. Furthermore, studies using a mouse skin colonization model demonstrated that CRISPR/Cas9 selectively reduced *staphylococci* colonization compared to other treatment conditions [410,411]. In another study, Ates et al. [412] showed that engineered CRISPR plasmids containing sgRNAs suppressed the *mecA*, gentamicin (*aacA*), and ciprofloxacin (*grlA*, *grlB*) resistance genes in MRSA strains, leading to altering the resistance profile and enhancing sensitivity to antibiotics. The CRISPR-Cas9 mediated plasmid-curing system (pCasCure) was employed to resensitize *Enterobacteriaceae* (CRE) to carbapenems. The results showed that pCasCure precisely cleaved *bla*_NDM_, *bla*_KPC_, and *bla*_OXA-48_ genes and targeted *rep*A, *rep*B, and *par*A on the pKpQIL plasmid to effectively clear the prevalent plasmid carrying the carbapenem- resistance gene and resensitize CRE, including *K. pneumoniae*, *E. coli*, *E. hormaechei*, *E. xiangfangensis*, and *S. marcescens* to carbapenem antibiotics [413]. Subsequently, Yosef et al. [414] applied CRISPR/Cas9 system to destroy plasmids carrying beta-lactamase genes *bla*_NDM-1_ and *bla*_CTX-M-15_ to kill extended-spectrum beta-lactamase (ESBL)-producing *E. coli*. In *E. coli* strain O157:H7 (EHEC), a conjugative CRISPR/Cas9 system targeting the mobile colistin resistance gene (*mcr-1*) eliminated not only drug-resistant plasmids and re-sensitized to antibiotics but also prohibited horizontal gene transfer after transformation with CRISPR/Cas9 plasmid [415]. Subsequently, Citorik et al. [416] demonstrated that the CRISPR/Cas system targeting *eae*, encoding virulence factor in *E. coli* O157:H7 (EHEC), caused a 20-fold reduction in viable cell counts. However, Rodrigues et al. [417] deployed the CRISPR/Cas9 system to selectively remove the erythromycin (ermB) and tetracycline (tetM) resistance genes in *E. faecalis* in vitro and in vivo. In vivo results showed a significant reducing the prevalence of antibiotic-resistant *E. faecalis* in the mouse gut after antibiotic treatment and intestinal infections caused by this bacterium [417].

More recently, Askoura et al. [418] reported that the CRISPR/Cas9 system targeting *sdiA* affected *S. enterica* biofilm formation, cell adhesion, and invasion. Additionally, the CRISPR/Cas-HDR approach was used to inhibit *E. coli* ATCC 25,922 biofilm formation by knockout genes involved in QS (*luxS*) and adhesion (*fimH*/*bolA*) [419]. Results showed that all mutant strains lacked extracellular polymeric substances (EPS) production compared to the wild-type strain; the noticed reduction of biofilm formation in Δ*fimH*, Δ*luxS*, and Δ*bolA* strains ranged between 75.39% and 84.17%. In addition, significantly higher adherence and cell aggregation, as well as biofilm formation on urinary catheters, were observed for wild-type strains [419].

Apart from CRISPR/Cas9, Kiga et al. [420] utilised CRISPR/Cas13a-based antibacterial nucleocapsids, CapsidCas13a, to effectively kill carbapenem-resistant *E. coli* and methicillin-resistant *S. aureus* by targeting antimicrobial resistance genes. On the other hand, the CRISPRi/dCas9 system was used to control the expression of the *wcaF* involved in the colanic acid synthesis, a key EPS component in *E. coli* biofilm formation. Depending on the level of the guide RNA (gRNA) controlled by a chemical inducer, *wcaF* expression was regulated by gRNA-dCas9 binding to the chromosomal *wcaF* locus; temporal induction showed different levels of biofilm thickness [421].

#### 4.2.2. sRNA Technologies

Growing evidence indicated that, like other bacterial processes, the integration of information by QS systems is regulated by noncoding small RNAs (sRNAs) called Qrr (quorum regulatory RNA), which are global regulators that act directly and indirectly to control gene expression via post-transcriptional mechanisms [152,422]. The role of Qrr-sRNA in modulating QS signalling has been described for the first time in *V. harveyi* and *V. cholerae* [423,424]. In the *Vibrionaceae*, the number of Qrr-sRNA is different between species, such as, for example, four Qrr-sRNAs in *V. cholerae* [425] and five Qrr-sRNAs in *V. harveyi* [426] and *V. *vulnificus** [427], respectively. In *V. cholerae*, Qrr1-4 sRNAs inhibit the expression of the *hapR* gene, which encodes a significant regulator of high-cell density behaviour that represses biofilm formation and virulence genes [426]. Therefore, targeting regulatory sRNAs may be another potential tool for blocking QS signalling by inhibiting the expression of genes involved in biofilm formation [152,428,429]. Mandin et al. [430] showed that the modulation of expression of several sRNAs, OmrR, OmrB, and McaS, leading to the change in cell motility, the production of curli, and the export of exopolysaccharides, results in the inhibition of *E. coli* biofilm formation. Also, the knockout of other sRNAs, Arc2, SdsR, GadY, and MicA affects biofilm formation and motility, although their mode of action remains elusive [430]. Metabolic engineering and the possibility to synthesize artificial RNAs of choice [431] create the opportunity for silencing any specific gene and, therefore, inhibit various steps of biofilm formation or enhance biofilm dispersal.

## 5. Prospects and Future Directions

Since the initial discovery of quorum sensing more than 40 years ago, the mechanistic understanding of various QS systems and appreciation for the importance of QS in the pathogenesis of many bacterial species have been expanded. Numerous studies confirmed that the QS system regulates biofilm formation in Gram-negative and Gram-positive bacterial strains. Bacterial biofilms, especially those formed by human pathogens, are relevant to chronic bacterial infections. Therefore, using QS-inhibiting agents is a promising therapeutic strategy targeting QS systems that is attracting attention in drug development. In recent years, many natural or synthetic QS-inhibiting strategies that effectively reduce biofilm formation have been developed, mainly thanks to the development of sophisticated microbiological techniques. Unfortunately, the potential risk of using all QQ strategies described above should also be mentioned [432]. Future studies in the therapeutic development of anti-virulence/antibiofilm strategies should proceed with care and caution to avoid the undesired fate currently associated with antibiotic development.

## Figures and Tables

**Table 1:** Quorum systems of selected Gram-negative and Gram-positive bacterial strains.

QS Molecules	Bacteria	QS System	Biological Function
3OC_12_-HSL	*Vibrio fischeri*	LuxI/LuxR	Induction of bioluminescence
3-OH-C4-HSL	*Vibrio harveyi*	LuxM/LuxN	Induction of bioluminescence, virulence production [6,7,147]
AI-2		LuxS/LuxP	
CAI-1		CqaA/CqsS	
3OC12-HSL	*Pseudomonas aeruginosa* *Pseudomonas fluorescent*	Las/LasR	Virulence (toxin A, elastase), biofilm formation, multiple extracellular enzymes, secondary metabolites (rhamnolipids) motility, exopolysaccharide production [52,54,77,84]
C4-HSL		RhlI/RhlR	
PQS		PqsABCD/PqsR	
HHQ		PqsH/PqsR	
3OC12-HSL		N.A./QscR	
C_6_-HSL; C_4_-HSL	*Sierratia liquefaciens* *Serratia* sp. *ATCC 39006* *Serratia proteamaculans* *Serratia marcescens SS-1*	SwrI/SwrR	Biofilm formation, swarming motility, protease, prodigiosin, and lipase production [55,56,59]
		LuxI/LuxR	
		SmaI/SmaR	
		SprI/SprR	
		SpnI/SpnR	
3OC_8_-HSL	*Escherichia coli*	N.A./SdiA	Motility, acid resistance, cell division, expression of virulence factors (antibiotic resistance), motility and biofilm formation, epithelial cell invasion [61,62,67]
AI-2		LuxS/LsrB	
AI-3/Epinephrine/		---/QseC	
Norepinephrine		Csrb/Csrc	
3OC_8_-HSL	*Salmonella Typhimurium*	N.A./SdiA	Motility, acid resistance [65]
C_8_-HSL C_6_-HSL 3OC_6_-HSl	*Yersinia* *pseudotuberculosis*	YpsR/YpsI YtbR/YpsI	Biofilm formation and motility, regulation of clumping motility [70,73]
AI-2	*Klebsiella pneumoniae*	LuxS/LsrB	Biofilm formation, expression of virulence factors, competence [62,63]
C8-HSL		---/---	
C12-HSL		---/---	
CSF	*Bacillus subtilis*	ComX/ComA	Competence and sporulation [96,102]
PapR	*Bacillus thuringiensis*	PapR/PlcR	Exoenzymes [107]
AIP	*Staphylococcus aureus*	AgrD/AgrC	Virulence production, exotoxins, and biofilm dispersal [95,97,99]
CSP	*Staphylococcus pneumoniae*	CmC/ComD	Competence, virulence production, autolysis [101,106]
GBAP	*E. faecalis*	FsrD/FsrC	Gelatinase, proteases production, adhesion, conjugation [111]
cCF10		CcfA/PrgX	

N.A.: not applicable; ---: not yet characterised.

**Table 2:** QQ mechanisms of alternatives to antibiotics with antimicrobial and antibiofilm activities.

Substance (s)/ Alternative (s)	Targeted Bacterial Pathogens	Mechanism of Action
QS Inhibitors—Plant-Derived Bioactive Compounds		
Clove	*E. coli*, *P. aeruginosa*, *S. aureus*, *K. pneumoniae*	Biofilm dispersal by downregulation *relA* expression, inhibition of AHL synthesis [363]
Garlic (ajoene)	*P. aeruginosa*	Reduction of rhamnolipid, protease synthesis by interaction with RhlR; reduction of C4-HSL activity [171]
Curcumin	*P. aeruginosa*	Inhibition of virulence gene expression [364]
Thymol	*E. coli*, *S. aureus*, *S. enteridis*, *P. aeruginosa*	Downregulation of *sarA expression*, increased membrane permeability, penetration of polysaccharide matrix, eradication of biofilm [365]
Oregano	*K. pneumoniae*, *P. aeruginosa*, *A. baumanii*	Increase membranę permeability, penetration polysaccharide matrix, eradication biofilm [365]
Cinnamon	*E. coli*, *MRSA*, *S. Typhimurium*, *S. enteridis*, *S. epidermidis*, *A. baumannii*	Reduction of rhamnolipid, proteases, alginate, and lipids; disruption of DNA, RNA, and protein synthesis; inhibition of biofilm formation by downregulation of *icaA* expression [366]
QQ enzymes		
Dispersin B	*S. aureus*, *E. coli*, *S. epidermidis*	Dispersal of biofilm by PNAG-hydrolysing glycosidase enzymes [367]
AiiA_B546_ lactonase	*Aeromonas hydrophila*	QS inhibition by hydrolysing of AHLs [368]
QsdA lactonase	*P. aeruginosa*	Hydrolysing of AHLs with an acyl chain ranging from C_6_ to C_14_ with or without a hydroxyl or oxo substitution on C_3_; reduction of rhamnolipid and elastase levels [369]
BpiB05 lactonase	*P. aeruginosa*	Reduction of motility, pyocyanin synthesis, and biofilm formation [369]
Lysostaphin	*MRSA*, *Streptococcus* sp., *S. epidermidis*	Degradation of cell wall by peptidoglycan hydrolysis [370]
Dnase NucB	*S. aureus*, *S. epidermidis*, *Staphylococcus salivarius*, *Staphylococcus constellatus*, *S. Staphylococcus lugdunesis*, *Staphylococcus anginosus*, *E. coli*, *Streptococcus intermedius*, *Micrococcus luteus*, *Bacillus subtilis*	Degradation mature biofilm formation [371]
Antimicrobial peptides		
Nisin A	*S. aureus*	Depolarization cell membranę [372]
Pilicides (FN075, BibC6, Ec240)	*E coli*	Inhibition of curli and Type I pili synthesis [373]
P1	*Streptococcus mutants*	Degradation EPS matrix [374]
Esculentin (1–12)	*P.aeruginosa*, *E. coli*, *S. aureus*, *MRSA*	Biofilm eradication [375]
Human β-defensin 3 (hBD-3)	*S. epidermidis*	Biofilm formation inhibition, downregulation of *icaA*, *icaD*, and *icaR* expression [376]
LL-37	*P. aeruginosa*, *A. baumanni*, *S. aureus*	Membrane disruption, reduction of swimming and swarming motilities, promotes twitching motility, downregulation genes of biofilm formation (*rhlA*, *rhhlB*), influence QS system [377]
Piscidin 3	*E. coli*, *S. aureus*, *A. baumannii*	Degradation eDNA [378]
1037	*P. aeruginosa*	Downregulation genes of biofilm formation, reduction of motilities, and swarming motilities [228]
Nal-P-13	*Porphyromonas gingivalis*	Downregulation genes of transport and binding proteins [379]
Antibodies		
EbpAFull, EbpANTD	*E. faecalis*	Blocking the interaction between EbpA and the host-inhibits biofilm formation [380]
Anti-IHFEc	*E. coli*, *H. influenzae*, *Burkholderia cenocepacia*	Inhibition biofilm formation [381]
Cam-03	*P. aeruginosa*	Blocking the attachment of *P. aeruginosa* to cultured epithelial cells, inhibiting the adherence or formation of denser biofilms [382]
TRL1068	*MRSA*	Disruption of biofilm formation [255]
Nanoparticles		
Silver (Ag)	*S. epidermidis*, MRSA, vancomycin-resistant *Enterococcus* (VRE), extended-spectrum beta-lactamase (ESBL)-producing organisms, MDR *E. coli*, *P. aeruginosa*, *K. pneumoniae*, carbapenem and polymyxin B-resistant *A. baumannii*, carbapenem-resistant *P. aeruginosa*, *E. coli*	Generate reactive oxygen species (ROS), stopping cytochrome chains, membrane damage, dissipation of proton gradients, and destabilisation of RNA and DNA [245,383]
Copper (Cu)	MDR *E. coli*, *A. baumannii*	Dissipation of cell membranes, generation ROS, lipid peroxidation, protein oxidation, and DNA degradation [383]
Zinc oxide (ZnO)	*Enterobacter aerogenes*, *E. coli*, *K. pneumoniae*, MRSA, *K. pneumoniae*, ESBL-producing *E. coli*	Generation of ROS, disruption of membranes, adsorption to the cell surface, and damage to lipids and proteins [384]
Gold (Au)	*MRSA*	Damage membranes and respiratory chains, decrease the binding between tRNA and ribosomes and formation of pores in the cell wall, inhibit ATPase activity [294]
Magnesium oxide (MgO)	*S. aureus*, *E. coli*	ROS generation, lipid peroxidation [383]
Probiotics		
*L. fermentum* TCUESC01, *L. plantarum* TCUESC02	*S. aureus*	Biofilm formation inhibition by alteration of the *ica* operon (*icaA*, *icaR*) [302,385]
*L. fermentum* KT998657	*P. aeruginosa PAO1*	Reduced biofilm forming, alteration of matrix and cell assembly, cell-cell interaction, and attachment to form biofilms [386]
*L. casei*, *L.reuteri*, *L. plantarum*, *L. salivarius*	*S. mutans*	Downregulation gene expression of acid tolerance, QS and EPS production, peroxide-dependent antimicrobial and antibiofilm activity (*L. salivarius*) [387]
*L. kefiranofaciens*, *L. plantarum*, *L. rhamnosus*, *L. johnsonii*	*S. mutans*, *S. sobrinus*	Downregulation gene expression of carbohydrate metabolism, regulatory biofilm, and adhesion proteins [388]
*L. plantarum*, *Pediococcus. acidilactici*	*S. aureus*	Downregulation gene expression of *cidA*, *icaA*, *dltB*, *agrA*, *sortaseA*, *sarA* [389]
Bacteriophages		
EFDG1	*E. faecium*, *E. faecalis*	Mature biofilm eradication [337]
vB_SauM_philPLA-RODI	*S. epidermidis*	Penetration biofilm, inhibition biofilm formation [340]
vB_PaeM_LS1	*P. aeruginosa*	Disruption biofilm formation [323]
Combined Therapies		
Curcumin/ciprofloxacin	*E. coli*, *K. pneumoniae*, *P. aeruginosa*, *E. faecalis*, *A. aureus*	QS inhibition [390]
Esculentin (1–21)/Au NPs	*P. aeruginosa*	Disruption membrane forming [391]
SAP-26/rifampicin	*S. aureus*	Mature biofilm eradication, hydrolysis bacterial wall [392]
Carvacrol/eugenol	*K. pneumoniae*, *S.aureus*, *P. aeruginosa*, *E. faecalis*	Increase in membrane permeability [365,393]
